# An Unusual Cause of Abdominal Pain: Three Lead Pellets within the Appendix Vermiformis

**DOI:** 10.1155/2015/496372

**Published:** 2015-05-28

**Authors:** Orhan Veli Ozkan, Vecdi Muderris, Fatih Altintoprak, Orhan Yagmurkaya, Omer Yalkin, Fehmi Celebi

**Affiliations:** ^1^Department of General Surgery, Faculty of Medicine, Sakarya University, Sakarya, Turkey; ^2^Department of General Surgery, Research and Educational Hospital, Sakarya University, Sakarya, Turkey

## Abstract

Most ingested foreign bodies usually pass out in the feces uneventfully. Complications such as intestinal perforation and bleeding usually occur with sharp, thin, stiff, long, and pointed objects. This case describes the management of three lead pellets within the appendix vermiformis. A 45-year-old male visited our clinic complaining of a 4-month history of abdominal pain. The patient inquiry revealed that he had eaten hunted rabbit meat on numerous occasions and had unintentionally ingested three lead pellets. Plain abdominal films and a barium enema showed foreign bodies in the right lower abdominal quadrant. Since the lead pellets were thought to have migrated extraluminally, they were removed through laparotomy under fluoroscopic guidance. An appendectomy was performed. Pathologically, three lead pellets were embedded in the appendix, which showed signs of intramucosal inflammation. Foreign bodies causing appendicitis are rare. However, if stiff or pointed objects enter the appendicular lumen, there is a high risk of appendicitis, perforation, or abdominal pain. An appendectomy was required to remove the ingested lead pellets in the appendix.

## 1. Introduction

In the United States, more than 100,000 patients annually are seen following the ingestion of foreign bodies. Most cases involve children. In adults, the ingestion of foreign bodies is uncommon and seen in individuals with mental disorders, prison inmates, or those with self-destructive (i.e., suicidal) behavior. Most ingested foreign bodies usually pass through the body in the feces uneventfully. Complications such as intestinal perforation and bleeding can occur with sharp, thin, stiff, long, or pointed objects. Less than 1% of these foreign bodies require surgical intervention [1, 2].

The localization of foreign bodies within the appendix is rare, with an estimated incidence of 0.0005% [3]. Various foreign bodies have been reported in the appendicular lumen, including pellets [4], needles [5], prosthetic teeth [6], and screws [7].

In this case, we describe the management of three lead pellets within the appendix vermiformis.

## 2. Case Presentation

**Figure 1:**
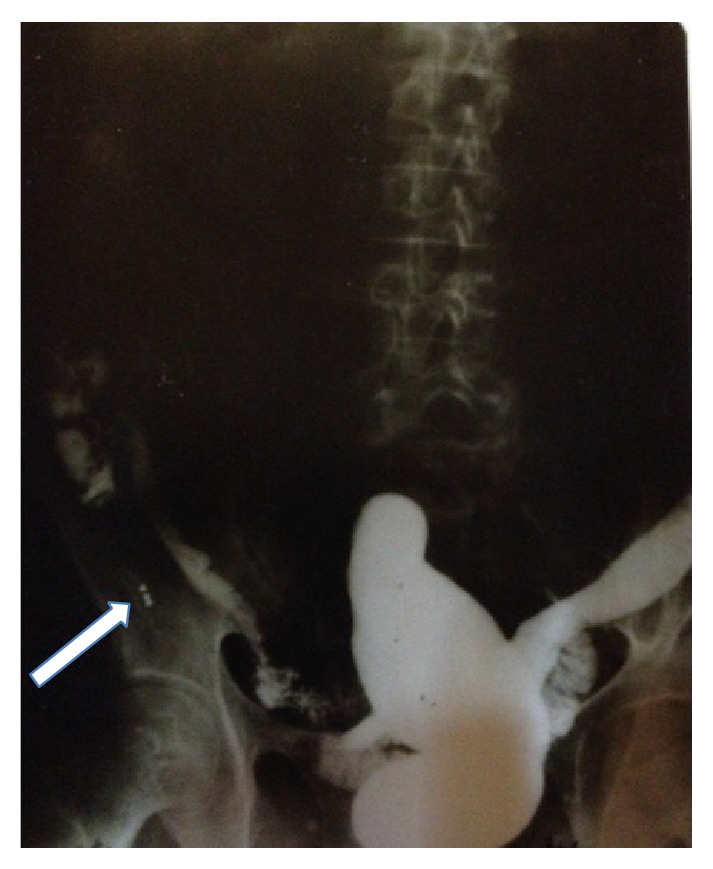
A barium enema shows opacities caused by three lead pellets within the appendix vermiformis (white arrow).

**Figure 2:**
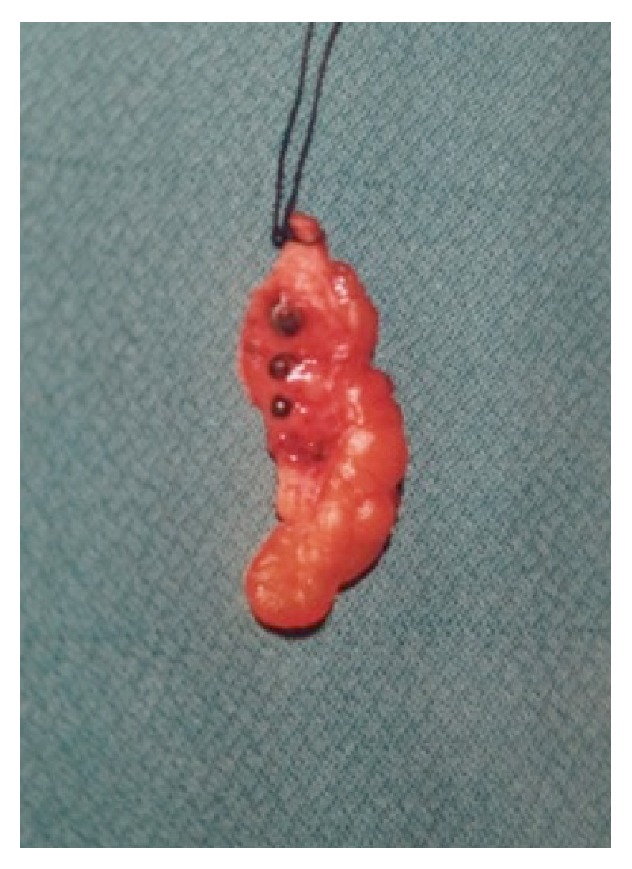
Three lead pellets within the appendix vermiformis.

A 45-year-old male presented to our clinic suffering from a four-month history of abdominal pain. Further questioning revealed that he had eaten hunted rabbit meat on numerous occasions and unintentionally ingested three lead pellets. The physical examination revealed tenderness in the right iliac fossa, but no signs of peritonitis. The lead levels were analyzed, since lead poisoning was considered in the differential diagnosis. All laboratory parameters, including lead levels, were normal, and the patient was afebrile. Plain abdominal films and a barium enema revealed foreign bodies in the right lower abdominal quadrant (Figure 1). Since the lead pellets were thought to have migrated extraluminally, we removed them with laparotomy under fluoroscopic guidance. The foreign bodies were found in the appendix vermiformis. An appendectomy was performed. Pathologically, three lead pellets were embedded in the appendix and there were signs of intramucosal inflammation (Figure 2). The patient was discharged on postoperative day 2 without complications. Three months later, he had recovered completely.

## 3. Discussion

Ingested foreign bodies are rarely found in the appendicular lumen. Claudius Amyand, a surgeon in Westminster Hospital, London, reported the first case in 1736, when operating on an 11-year-old patient with a stubborn fecal fistula; during surgery, he detected that the appendix was perforated by a “pin” within the hernia sac [8]. Ingested foreign bodies can remain immobile within the appendix for a long time without causing an inflammatory response or can cause an inflammatory reaction without causing perforation. Sharp, stiff foreign bodies, such as sewing needles and metallic screws, or even shotgun pellets, can cause acute appendicitis. The clinical findings of foreign bodies localized within the appendix range from asymptomatic to those of perforated appendicitis [9].

Ingested lead pellets localized within the appendix cause symptoms and findings depending on the clinical conditions they produce. Some cases are asymptomatic, while others cause acute appendicitis, leading to symptoms and findings of an acute abdomen. If many are present, they can lead to lead poisoning. In most of the patients the retained lead pellets are not removed because of difficulty during the operation. Indications for bullet removal include bullets found in the joint, cerebrospinal fluid, or the globe of the eye, pellets leading to impingement on a nerve or a nerve root, and bullets lying in the lumen of a vessel. Lead poisoning is also rare indication [10]. Lead poisoning, also known as plumbism and saturnism, is a type of metal poisoning caused by increased levels of lead in the body. Lead poisoning may be acute or chronic, the latter being much more common. Presentations of adult lead poisoning range from nonspecific symptoms to acute encephalopathy. Cox and Pesola [11] define lead-shot accumulation in the appendix vermiformis of an Alaskan native, which was likely caused by the ingestion of shotgun-culled waterfowl. A potential risk exists for lead intoxication in case of many lead shots in the appendix. A few cases of lead intoxication in the literature have been reported. Therefore, European Countries such as Denmark and Netherlands have banned lead for hunting and suggest that lead shots include tin, steel, bismuth, and tungsten, owing in part to the concern regarding lead toxicity from this practice [12–15]. Our patient suffered from abdominal pain, but his blood lead levels were normal. We concluded that long-term localization of the lead pellets within the appendix resulted in inflammation, which caused the patient's complaints.

The diagnosis of lead pellets localized within the appendix lumen is based on a good history, and their radiopacity makes them easy to detect radiologically. The history should include questions about the patient's occupation and eating habits, to identify the etiology of abdominal pain. In our case, the patient's vocation (i.e., hunting rabbits) helped us to identify the foreign bodies seen radiologically. Multiple lead pellets were reported in a patient who shot and ate a pigeon [16], while 57 lead pellets were removed from the appendix of an 8-year-old child who ate geese killed with lead shots [4].

Endoscopic removal is recommended for treating ingested foreign bodies within the appendix, provided they are visible from the cecum during colonoscopy. If impossible, it is recommended that the foreign bodies be localized under endoscopic view and removed via laparoscopic appendectomy [2, 5, 16]. When it is impossible to remove the foreign bodies with these methods, a laparotomy and appendectomy should be performed [17, 18]. In our case, the foreign bodies were localized in the right lower quadrant under fluoroscopic guidance and an appendectomy was performed during laparotomy, since the foreign bodies were outside the lumen. The patient recovered fully after the appendectomy.

A lead pellet in the appendix lumen is an extremely rare condition that can lead to abdominal pain, appendicitis, and lead poisoning. Therefore, the ingested lead pellet should be removed surgically with an appendectomy.
